# Single-Tooth Morse Taper Connection Implant Placed in Grafted Site of the Anterior Maxilla: Clinical and Radiographic Evaluation

**DOI:** 10.1155/2014/183872

**Published:** 2014-11-05

**Authors:** Francesco Guido Mangano, Piero Zecca, Fabrizia Luongo, Giovanna Iezzi, Carlo Mangano

**Affiliations:** ^1^Department of Surgical and Morphological Science, Dental School, University of Insubria, 21100 Varese, Italy; ^2^ITEB Research Centre, University of Insubria, 21100 Varese, Italy; ^3^Private Practice, 00193 Rome, Italy; ^4^Department of Medical, Oral and Biotechnological Sciences, Dental School, University G. d'Annunzio, 66100 Chieti, Italy

## Abstract

The aim of this study was to achieve aesthetically pleasing soft tissue contours in a severely compromised tooth in the anterior region of the maxilla. For a right-maxillary central incisor with localized advanced chronic periodontitis a tooth extraction followed by reconstructive procedures and delayed implant placement was proposed and accepted by the patient. Guided bone regeneration (GBR) technique was employed, with a biphasic calcium-phosphate (BCP) block graft placed in the extraction socket in conjunction with granules of the same material and a resorbable barrier membrane. After 6 months of healing, an implant was installed. The acrylic provisional restoration remained in situ for 3 months and then was substituted with the definitive crown. This ridge reconstruction technique enabled preserving both hard and soft tissues and counteracting vertical and horizontal bone resorption after tooth extraction and allowed for an ideal three-dimensional implant placement. Localized severe alveolar bone resorption of the anterior maxilla associated with chronic periodontal disease can be successfully treated by means of ridge reconstruction with GBR and delayed implant insertion; the placement of an early-loaded, Morse taper connection implant in the grafted site was effective to create an excellent clinical aesthetic result and to maintain it along time.

## 1. Introduction

Advanced chronic periodontitis is a significant reason for tooth loss in adult patients [1]. The loss of an anterior tooth compromises the patient's aesthetics and has major detrimental implications for the subject, since it significantly affects his/her social integration and quality of life [1–3]. Single implant is a valid treatment procedure, to restore aesthetics in the anterior maxilla, at least in situations where an adequate bone volume is present [3–5]. However, a severely compromised tooth in the maxillary anterior region poses a great challenge to implant therapy. As such, prior to implant placement, an advanced bone defect needs reconstructive procedures to restore the original anatomy, for a predictable long-term aesthetic outcome [6, 7]. A key prerequisite for a positive aesthetic outcome in implant treatment is an adequate three-dimensional (3D) osseous volume of the alveolar ridge, including an intact facial bone wall of sufficient thickness and height [6–8]. After the extraction of a severely compromised tooth in the anterior maxilla, a local reduction of the vertical and horizontal dimensions of the alveolar ridge may occur, leaving the patient with insufficient bone to allow implant placement [6–8]. In particular, a deficiency of facial bone anatomy has a negative impact in the anterior region, leaving the patient with an undesirable aesthetic situation [8–10]. In these contexts and following tooth extraction, regenerative techniques have been recommended to allow for ridge augmentation, which improves soft and hard tissue volume for the implant placement [11, 12]. Over the years, a number of ridge reconstruction techniques have been proposed, such as buccolingual expansion with osteotomes, screws ridge-splitting techniques, augmentation with autografts, allografts, and xenografts, as well as guided bone regeneration (GBR) [11–13]. The surgical technique of GBR is a method for achieving bone regeneration by creating a secluded anatomic site using barrier membranes, with or without a biomaterial scaffold, to promote healing [11, 13, 14]. GBR technique uses different types of membranes to maintain the space and to protect the blood clot formed in the bony defect (such as socket voids created by extractions). With these devices it is possible to prevent the migration of the undesired epithelial cells and connective tissue fibroblasts from the adjacent tissue into the defects [13, 14]. As a consequence, osteogenic cell populations from the native bone are prompted to stimulate new bone formation in the bone defect [13, 14]. Various grafting materials have been used to fill the gap and to promote bone healing in GBR [11–14]. Among these, a well-known synthetic bone substitute is bioceramics, such as biphasic calcium phosphate (BCP), which have been used in several oral grafting procedures [13, 15, 16]. BCP generally consists of 70% beta-tricalcium phosphate and 30% hydroxyapatite. It is a biocompatible osteoconductive alloplast with the ability to induce mesenchymal cells to differentiate toward osteoblasts, so that it is considered an optimal scaffold material for bone tissue engineering [13, 15, 16]. Histological studies in both animals [17] and humans [13, 15–18] found that BCP has a resorption rate that enabled new bone formation, without interfering with the bone matrix. BCP has been successfully used in the clinical scenario to fill bone defects and in sinus augmentation procedures [15, 16, 18]. However, there are still no clinical studies that evaluate the outcome of dental implants placed in the anterior maxilla, in aesthetically sensitive sites regenerated using BCP as grafting material. In the last few years, cone beam computed tomography (CBCT) has become a commonly accepted diagnostic tool, as it offers extremely accurate 3D diagnostics allowing for small fields-of-view (FOV), good image quality, and low radiation doses [9, 13]. Today, CBCT is recommended as the imaging method of choice for the assessment of dental implant sites [9, 13]. The objective of this report is, therefore, to document the clinical and radiographic outcome of an early-loaded single-tooth, Morse taper connection implant placed into an anterior maxilla site grafted with BCP, with an emphasis on the aesthetic result.

## 2. Case Presentation 

**Figure 1:**
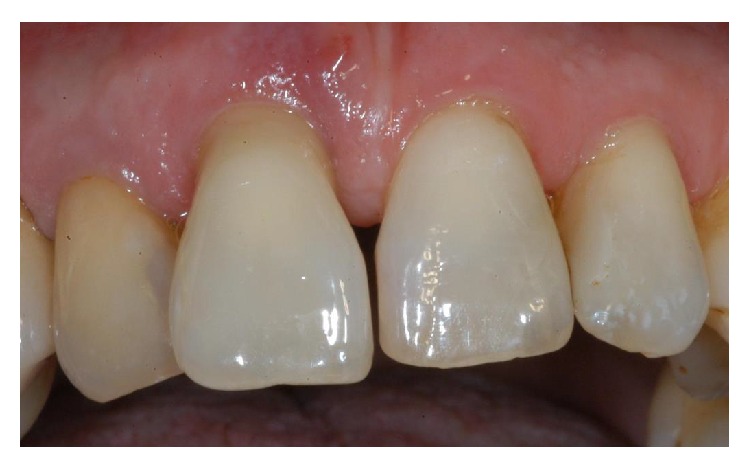
The right-maxillary central incisor was considerably extruded. The patient complained of mobility and slight localized pain during function.

**Figure 2:**
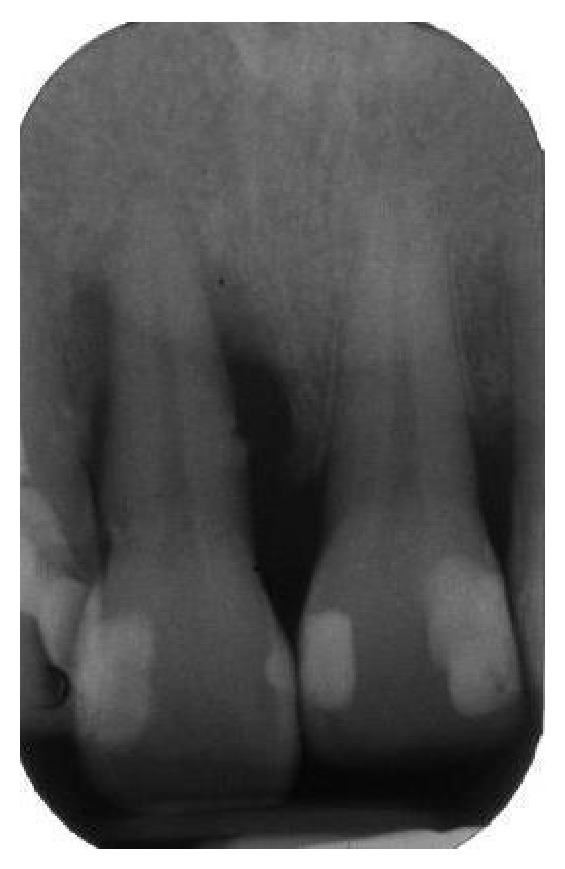
The periapical radiograph revealed a localized, severe bone resorption affecting the tooth.

**Figure 3:**
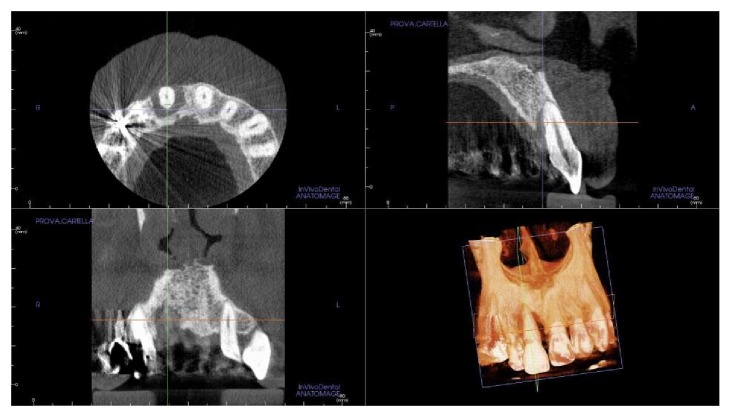
The small field-of-view (FOV) cone-beam computed tomography (CBCT) with three-dimensional (3D) reconstruction by means of an implant navigation software (Invivo Dental 5, Anatomage, San Jose, CA, USA) confirmed the presence of an advanced, localized bone resorption affecting the right-maxillary central incisor.

**Figure 4:**
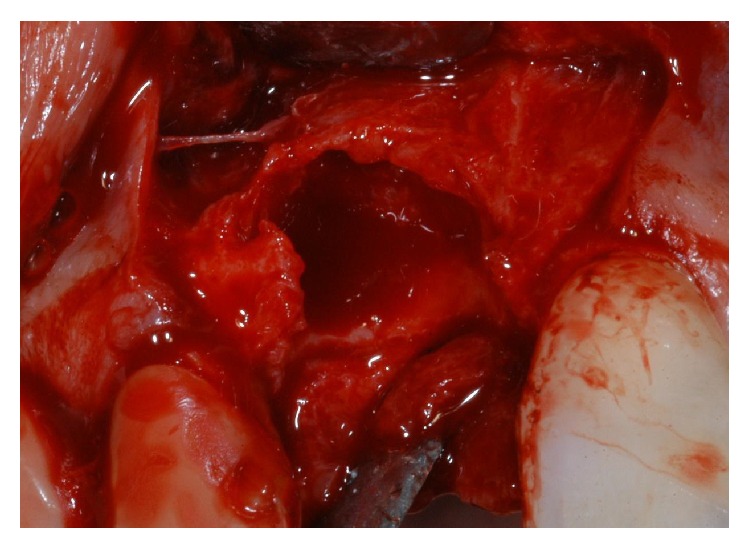
Immediately after tooth extraction, the alveolar bone review depicted a huge bone defect (>8 mm) with loss of a considerable amount of buccal bone.

**Figure 5:**
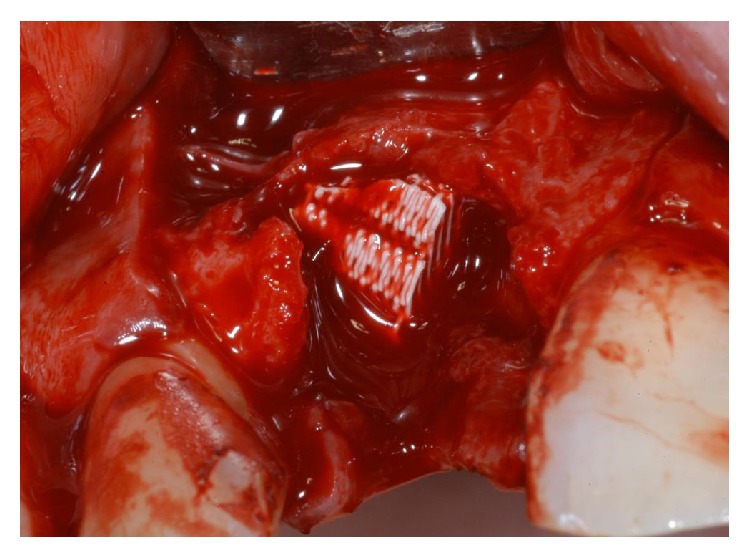
The socket was filled with a synthetic, micromacroporous biphasic calcium-phosphate (BCP) block.

**Figure 6:**
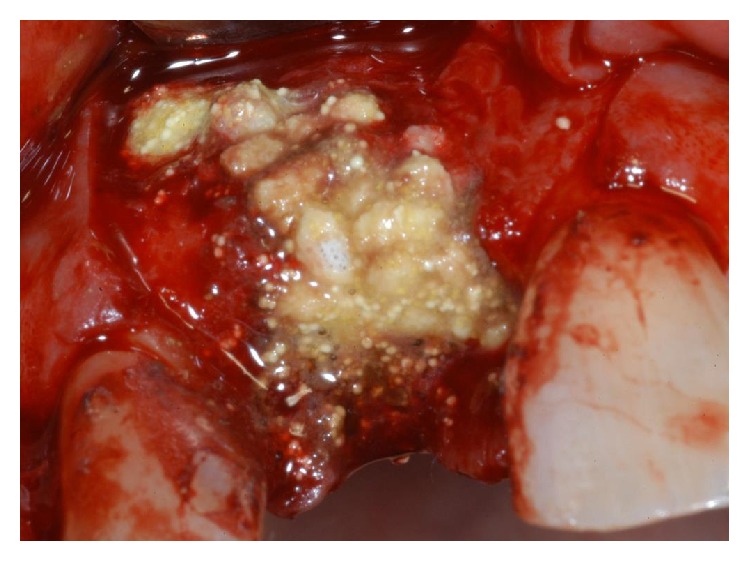
Granules of synthetic, micromacroporous BCP, mixed with tetracycline powder, were applied to completely fill and cover the bone defect.

**Figure 7:**
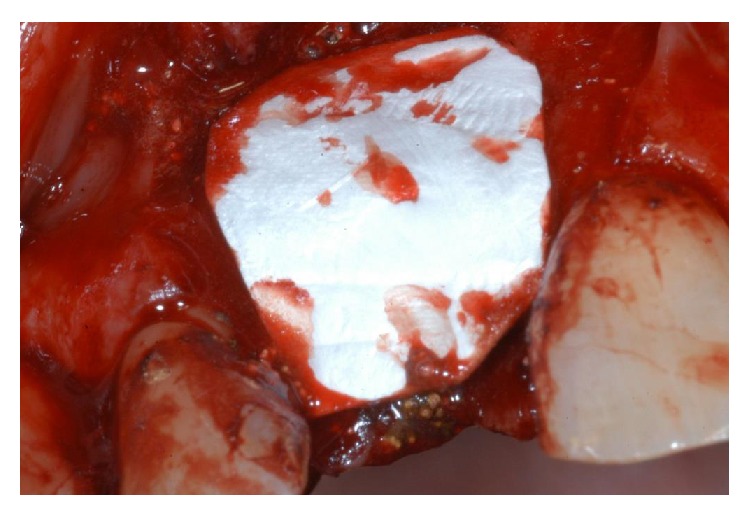
An absorbable collagen membrane was placed over the graft, covering all the defect and adjacent bone borders.

**Figure 8:**
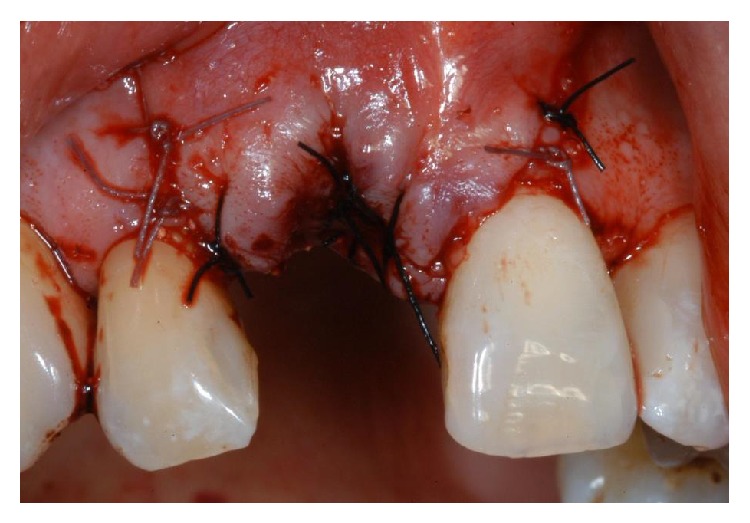
The flap was sutured in position.

**Figure 9:**
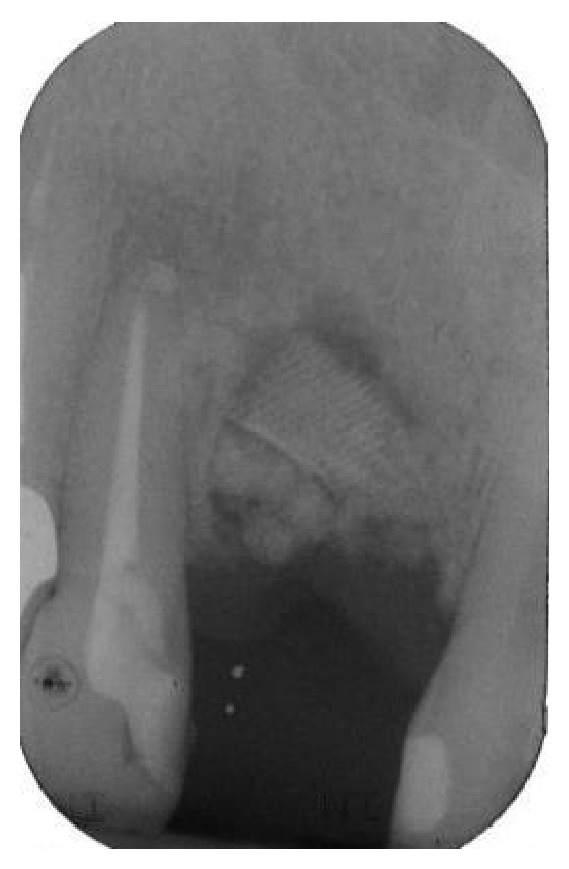
A postoperative periapical radiograph was taken to confirm the filling of the postextraction socket.

**Figure 10:**
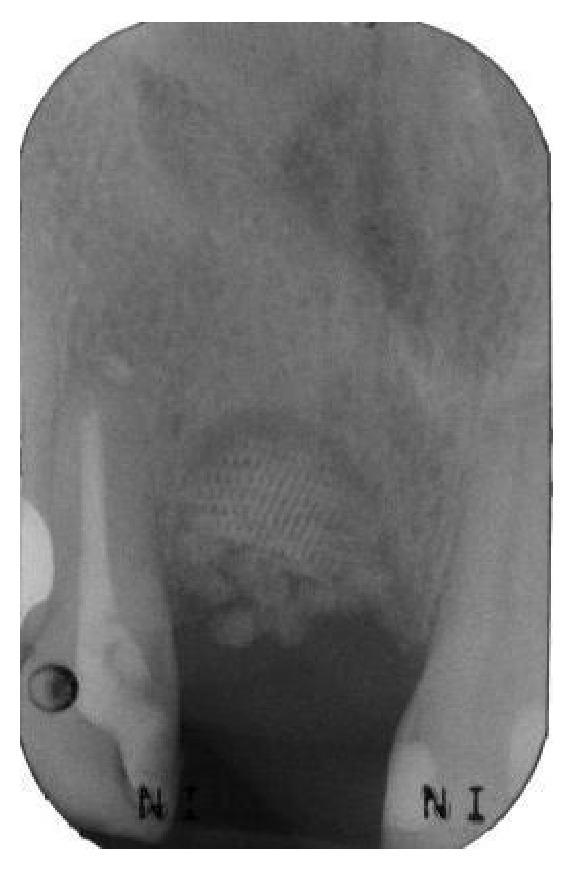
After 6 months of uneventful healing, a periapical radiograph showed good integration of the material used for regeneration.

**Figure 11:**
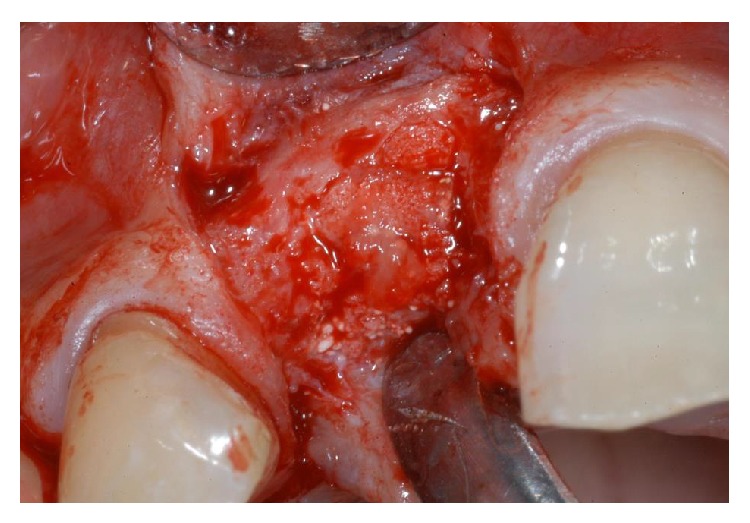
Six months after the grafting procedure, the patient showed great bone augmentation, confirming the possibility to place a dental implant in the proper position.

**Figure 12:**
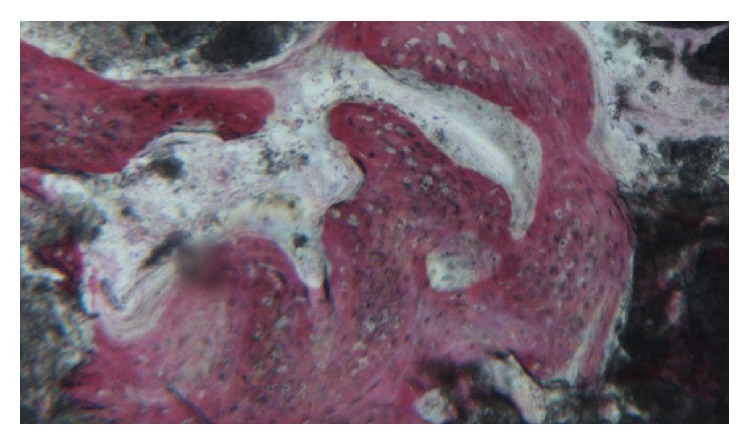
Histological evaluation revealed compact mature bone undergoing remodeling, marrow spaces, and newly formed trabecular bone surrounded by several residual biomaterial particles. The newly formed bone appeared well organized. Close to the porous BCP particles, new bone formation was observed, with newly formed osteoid matrix undergoing mineralization.

**Figure 13:**
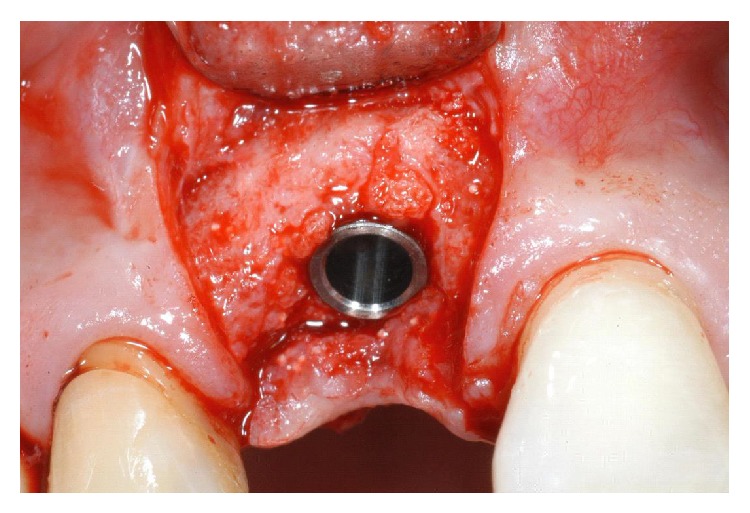
The 4.1 × 12 mm Morse taper connection implant immediately after placement in the regenerated area.

**Figure 14:**
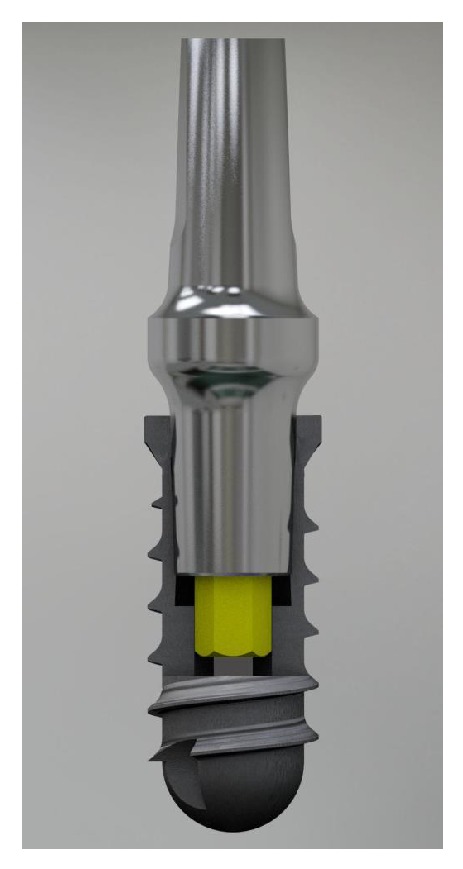
The implant used in this report is characterized by a cone Morse taper interference-fit (TIF) locking-taper combined with an internal hexagon. The Morse taper presents a taper angle of 1.5°.

**Figure 15:**
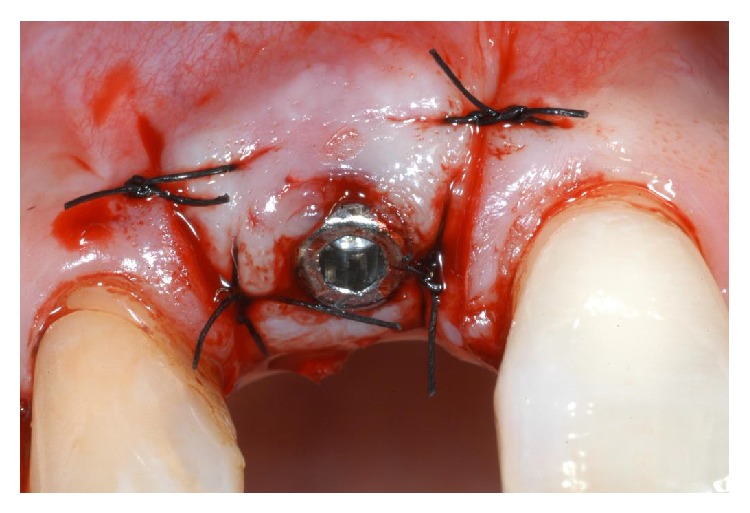
Immediately after implant placement, a healing abutment was connected to the implant. The mucosal flap was adjusted to the healing abutment and then sutured in position.

**Figure 16:**
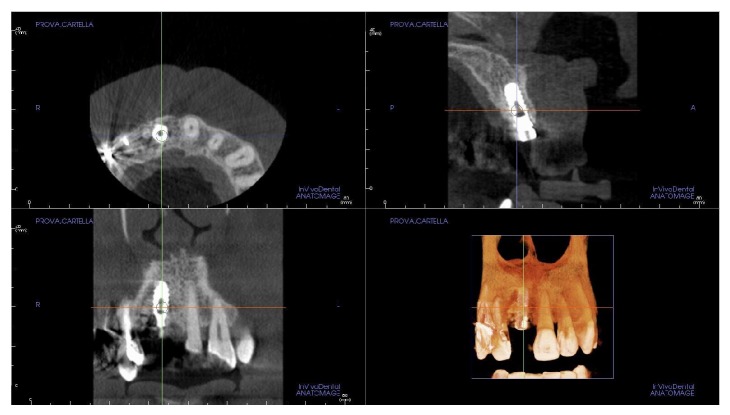
A second small FOV CBCT with 3D reconstruction confirmed optimal implant placement in the regenerated bone.

**Figure 17:**
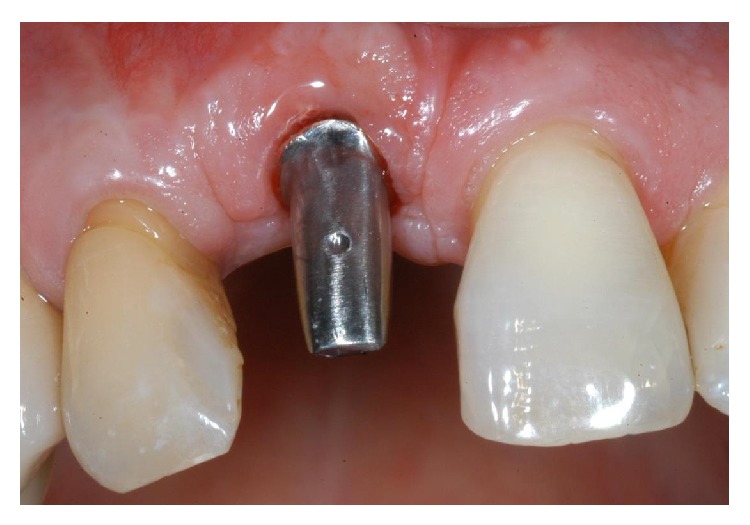
Two weeks after implant placement, a standard prefabricated, prepared, and finished titanium abutment was placed and activated.

**Figure 18:**
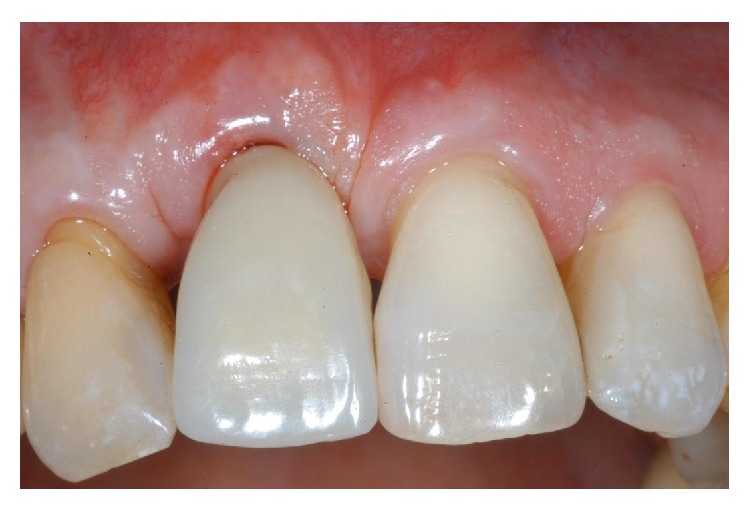
The acrylic resin provisional restoration in position.

**Figure 19:**
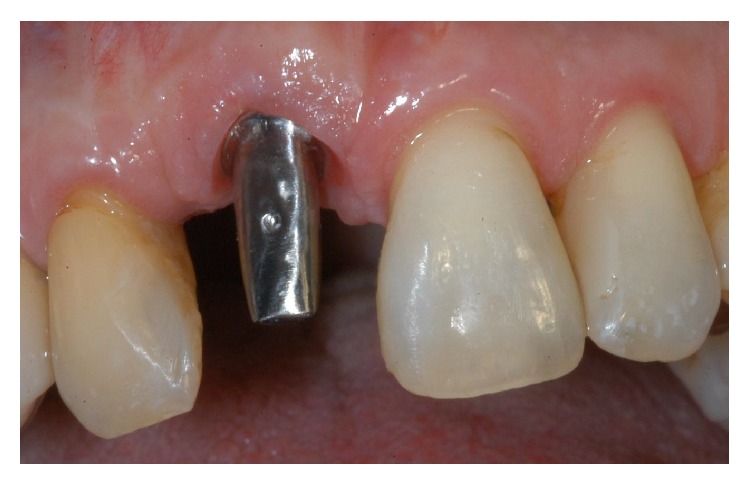
Three months after the placement of provisional restoration, the patient showed remarkable healing and conditioning of the soft tissues. The gingiva appeared with an excellent color and texture, while the facial mucosa curvatures began to outline a proper and harmonious design.

**Figure 20:**
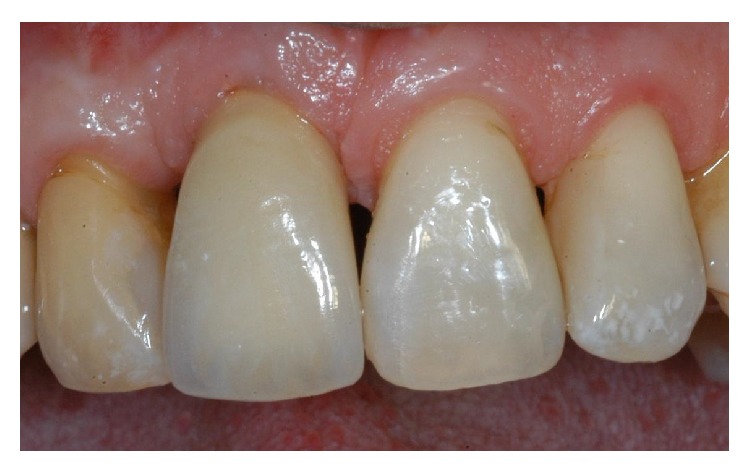
The definitive, ceramo-metallic restoration in position. The prosthetic restoration showed a good aesthetic integration: patient's smile aesthetics was improved and a satisfying harmony and symmetry with the contralateral tooth was achieved.

**Figure 21:**
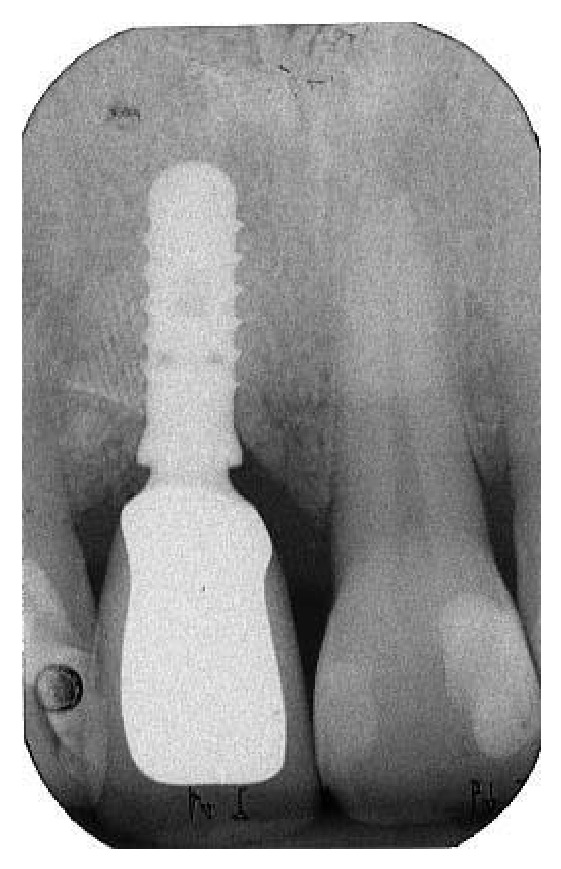
Periapical radiograph showing the definitive restoration seated in position.

**Figure 22:**
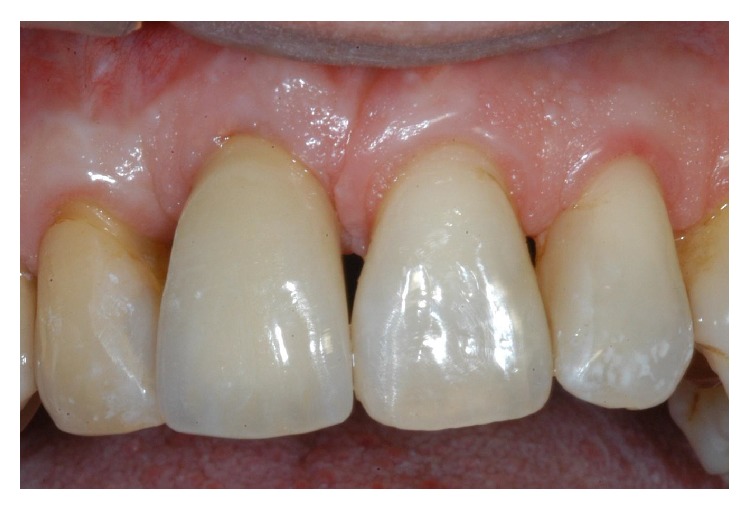
Two-year control. The implant was in function, showing an excellent aesthetic integration; clinical examination showed absence of gingival recession, no probing pocket depths, and no bleeding on probing or suppuration.

**Figure 23:**
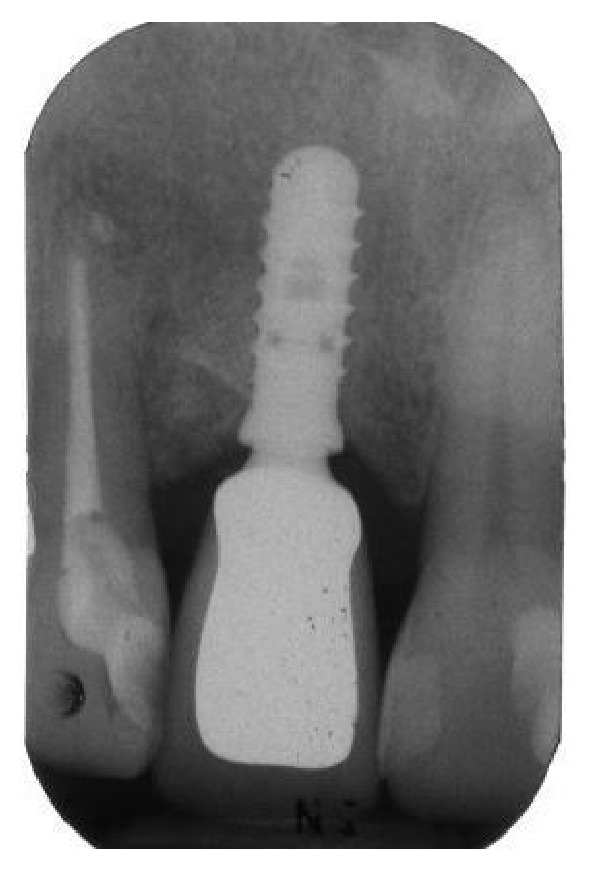
Periapical radiograph taken two years after implant placement.

**Figure 24:**
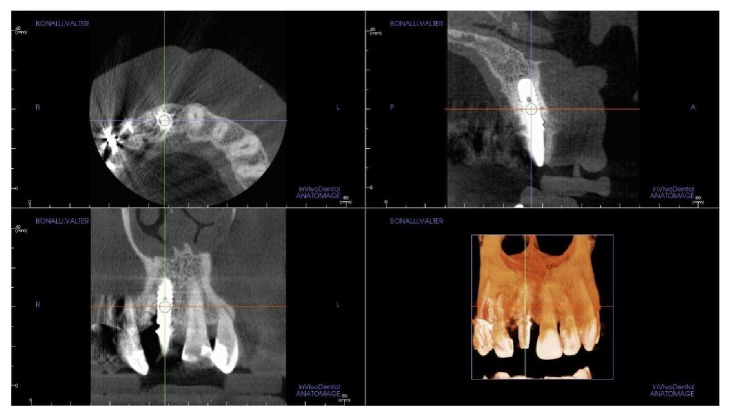
A third small FOV CBCT with 3D reconstruction confirmed excellent osseointegration of the implant with unchanged peri-implant marginal bone levels, indicating that the treatment proposed was able to restore the functional and aesthetic parameters.

**Figure 25:**
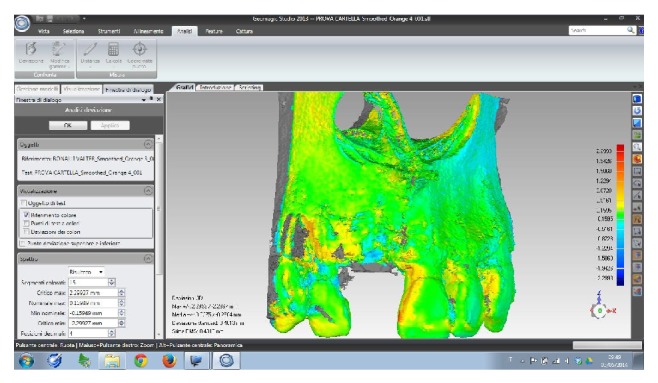
Overlapping of digital images. The DICOM (digital imaging and communication in medicine) files of the obtained CBCT datasets, 6 months and 2 years after grafting with synthetic, micromacroporous biphasic calcium-phosphate, were converted into a surface mesh model with digital imaging software (Mimics, Materialise, Leuven, Belgium). The two surface mesh models were then superimposed and rigidly aligned with anatomical landmarks, with the aid of a software for the overlapping of digital images (Geomagic Studio, Morrisville, NC, USA). The distance between the 2 surface meshes was presented as color-coded graded figures (blue: tissue loss; orange/red: tissue apposition; green/yellow: little or no modifications) to identify zones of bone resorption. In the frontal view, little or no buccal bone loss was evidenced, confirming the stability of the regenerated bone along time.

**Figure 26:**
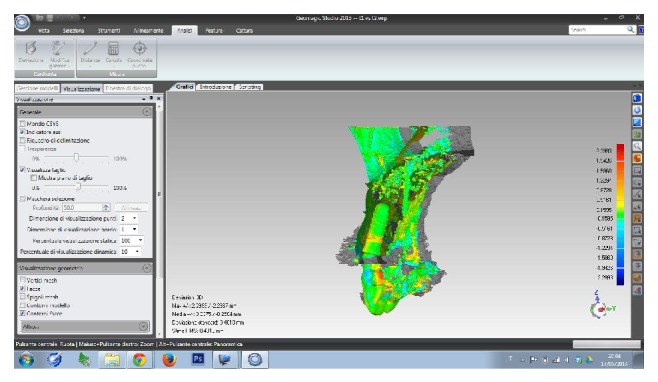
Overlapping of digital images, sagittal view: the stability of the regenerated bone along time was confirmed.

A 60-year-old male patient, nonsmoker, nonbruxist, and without any history of systemic disease, was referred to a single private practice (Gravedona, Como, Italy) for the evaluation and treatment of his right-maxillary central incisor. His chief complaints were of mobility and slight localized pain during oral function. The patient reported episodes of swelling in the right central incisor area. The tooth was considerably extruded (Figure 1). Vitality tests on tooth (cold) were positive. Clinical examination revealed poor oral hygiene, localized gingival recessions, and thick gingival tissues. Probing pocket depth (PPD) was measured using a light probing force (approximately 25 g), with a conventional periodontal probe (PCP-UNC 15, Hu-Friedy Manufacturing, Chicago, IL, USA) at 4 sites per tooth (mesially, mid-buccally, distally, and mid-lingually). PPD ranging from 3 to 6 mm were registered in all other teeth; for the right-maxillary central incisor, a localized 12 mm PPD with bleeding on probing and suppuration was detected at the buccal face, while PPD of 9, 8, and 9 mm were detected at the mesial, distal, and palatal faces, respectively. A periapical radiograph was taken, revealing a localized, severe bone resorption affecting the right-maxillary central incisor (Figure 2). For a better investigation of the local anatomy, CBCT datasets of the failing tooth were acquired using a modern cone beam scanner (CS9300, Carestream Health, Rochester, NY, USA). A small, 5 × 5 cm FOV was selected, with a voxel size of 90 *μ*m in order to obtain the best image resolution for the selected area, at lower radiation dose. CBCT dataset was then transferred to an implant navigation software (Invivo Dental 5, Anatomage, San Jose, CA, USA) to perform a 3D reconstruction of the anterior maxilla. The CBCT with 3D reconstruction confirmed the presence of the advanced, localized bone resorption (Figure 3). Based on clinical and radiographic examinations, tooth extraction followed by reconstructive procedures and delayed implant placement was proposed and accepted by the patient. Information was given to the patient regarding alternative treatment options (fixed partial denture on natural teeth). The patient received thorough explanations about the planned treatment and its potential risks and complications and signed a written informed consent form. Before the start of the treatment, for aesthetic reasons, an alginate impression was taken and a plaster cast was made, to fabricate a resin-bonded fixed partial denture as interim prosthesis. In addition, a diagnostic wax-up for the missing teeth structure was done, to provide the clinician with a better understanding of the patient's prosthetic needs and to ascertain the aesthetic outcome. Two weeks before extraction, the patient underwent a periodontal treatment, involving instructions and reinforcement in his oral hygiene efforts, followed by a scaling and root planning in the entire dentition. Surgery was performed under a local anaesthesia, obtained by infiltrating articaine 4%, containing 1 : 100,000 adrenaline (Ubistesin; 3M Espe, St. Paul, MN, USA). An intrasulcular incision was done, connected by two vertical releasing incisions and a full-thickness flap was reflected. The hopeless tooth was extracted avoiding any movement that might damage the residual buccal bone plate. Once the tooth was removed, the socket was thoroughly debrided with curettes and irrigated with sterile saline. The adjacent teeth were scaled and planed. The socket walls were then carefully probed, in order to assess the presence of any fenestration or dehiscence defects. The alveolar bone review depicted a huge bone defect (>8 mm) with loss of a considerable amount of buccal bone (Figure 4). In particular, the residual buccal bone wall was thin (width <2 mm). A technique for ridge reconstruction was adopted. A synthetic, micromacroporous biphasic calcium-phosphate block (Biocer, Biocer Entwicklungs GmbH, Bayreuth, Germany) was placed into the socket (Figure 5); then, granules of the same material were applied to completely fill the bone defect. The granules were mixed with tetracycline powder (Ambramicina; Scharper Spa, Sesto San Giovanni, Italy) to obtain a local antibiotic effect, and this mixture was moistened with physiological saline solution so that the composition could be more easily moulded to cover the defect (Figure 6). Finally, an absorbable collagen membrane (EZ Cure, Leone Implants, Florence, Italy) was placed over the graft, covering all the defect and adjacent bone borders (Figure 7). The flap was moved coronally to completely cover the membrane barrier and sutured in position by means of interruptedsutures (Supramid; Novaxa Spa, Milan, Italy) (Figure 8). A postoperative periapical radiograph was taken to confirm the filling of the postextraction socket (Figure 9). The patient was prescribed oral antibiotics, 2 g of amoxicillin/clavulanic acid each day for 6 days (Augmentin; Glaxo-Smithkline Beecham, Brentford, UK). Postoperative pain was controlled by administering 100 mg nimesulide (Aulin; Roche Pharmaceutical, Basel, Switzerland) every 12 h for 2 days, and detailed instructions about oral hygiene were given, including mouth rinses with 0.12% chlorhexidine (Chlorexidine; OralB, Boston, MA, USA) administered for 7 days. An interim prosthesis was delivered by using an adhesive system to attach to the adjacent teeth. This prosthesis was key in achieving an acceptable aesthetic outcome. The patient was seen two weeks after surgery for suture removal. He had mild swelling for 2-3 days after surgery, but no further discomfort during the healing period. Regular postoperatively examinations were performed at 3-month intervals and included oral hygiene instructions and professional plaque control. After 6 months of uneventful healing, the placement of a dental implant was planned, to restore aesthetics and function. A periapical radiograph was taken, showing an apparent good integration of the material used for regeneration (Figure 10). Again, local anaesthesia was obtained by infiltrating articaine 4% containing 1 : 100.000 adrenaline. Exposure of the regenerated ridge was achieved with a crestal incision and two lateral releases. Care was taken to preserve the papillae of the adjacent teeth. A mucoperiosteal flap was elevated. The patient showed great bone augmentation, confirming the possibility of placing a dental implant in the proper position (Figure 11). The osteotomy started with a 2 × 10 mm trephine bur, which was used to retrieve a bone core (approximately 2 × 6 mm) biopsy at the site of implant placement, via a transcrestal path, under saline solution irrigation. The bone core biopsy was retrieved with the aim of performing a histologic evaluation of the augmented bone. The biopsy was immediately stored in 10% buffered formalin and was subsequently processed (Precise 1 Automated System, Assing, Rome, Italy) to obtain thin ground sections. The specimens were dehydrated in an ascending series of alcohol rinses and embedded in glycol methacrylate resin (Technovit 7200 VLC, Heraeus Kulzer GmbH & Co., Wehrheim, Germany). After polymerization, the specimens were sectioned lengthwise along the longer axis, using a high-precision diamond disk saw, to about 150 *μ*m and ground down to about 30 *μ*m. Two slides were obtained from each specimen. The slides were stained with basic fuchsin and toluidine blue and the histologic evaluation was performed. The specimens were made of preexisting, compact mature bone undergoing remodeling, marrow spaces, and newly formed trabecular bone surrounded by several residual biomaterial particles. The newly formed bone appeared well organized. Close to the porous BCP particles, new bone formation was observed, with newly formed osteoid matrix undergoing mineralization (Figure 12). The preparation of implant site progressed with spiral drills of increasing diameter (2.8 and 3.5 mm, to place an implant with 4.1 mm diameter) under constant saline irrigation. The socket preparation was deepened beyond the alveolar apex, engaging the native apical bone, in order to obtain an optimal implant stability. A 4.1 × 12 mm implant (Leone Implants, Florence, Italy) was installed in the prepared site, using 20 rpm at 40 Ncm torque (Figure 13). This implant is characterized by a cone Morse taper interference-fit (TIF) locking-taper combined with an internal hexagon. The Morse taper presents a taper angle of 1.5° (Figure 14). The implant was positioned at the bone crest level, 2 mm apically to the cementoenamel junction of the left maxillary central incisor. Care was taken to ensure the correct 3D position of the implant and to keep a safe distance from the reconstructed buccal bone wall. A nonsubmerged, single-stage approach was followed. Immediately after implant placement, a healing abutment was connected to the implant. The mucosal flap was adjusted to the healing abutment and then sutured in position (Figure 15). The patient underwent a second 5 × 5 cm FOV CBCT examination with a voxel size of 90 *μ*m: the 3D reconstruction confirmed the optimal implant placement in the regenerated bone (Figure 16). The patient was seen on a weekly basis during the first 2 weeks. At the first control visit, 7 days after the surgery, a clinically healthy marginal area was present and no postoperative pain or swelling was reported. There was no bleeding or wound infection. After 14 days, sutures were removed; the healing abutment was removed and an impression coping was connected to the implant. Impressions were taken, using a vinylpolysiloxane material (EliteHd Plus, Zhermack, Badia Polesine, Italy). One week later, a standard prefabricated prepared and finished titanium abutment was placed and activated (Figure 17), and the acrylic resin provisional restoration was provided (Figure 18) and cemented with zinc-eugenol oxide cement (Temp-Bond, Kerr, Orange, CA, USA). Occlusion was checked using standard occluding papers (Bausch Articulating Papers, Bausch Inc, Nashua, NH, USA). The provisional restoration was carefully evaluated for proper occlusion; after that, it was polished with abrasive points. The acrylic provisional restoration remained in situ for 3 months: it was used to monitor the implant stability under a progressive load and to obtain a good soft-tissue healing around the implant before fabrication of the definitive restorations. At the end of this period, the patient showed remarkable healing of the soft tissues, and the gingiva showed an excellent color and texture. It also began to outline the proper and harmonious design of the facial mucosa curvatures, which were conditioned by the provisional restoration (Figure 19), so that the definitive, ceramo-metallic restoration could be provided (Figure 20) and cemented with zinc-eugenol oxide cement. The prosthetic restoration showed a good aesthetic integration: the patient's smile aesthetics was improved and a satisfying harmony and symmetry with the contralateral tooth were achieved. A periapical radiograph was taken to check definitive restoration seating (Figure 21). Two years after implant placement, the implant was stable and in function, with no clinical issues; clinical examination showed absence of gingival recession, no probing pocket depths, and no bleeding on probing or suppuration (Figure 22). Periapical radiographic evaluation revealed a stable alveolar bone gain, especially in the vertical dimension (Figure 23). The definitive restoration was removed and a new 5 × 5 cm FOV CBCT examination with a voxel size of 90 *μ*m, combined with 3D reconstruction, was taken. It confirmed excellent osseointegration of the implant with unchanged peri-implant marginal bone levels, indicating that the treatment proposed was able to restore the functional and aesthetic parameters (Figure 24). Finally, in order to evaluate precisely the hard tissue stability along time, the data of the second (6 months after grafting) and the third (2 years) CBCT were segmented by digital imaging software (Mimics, Materialise, Leuven, Belgium). Based on the result of segmentation, according to Chappuis and colleagues [9] a surface mesh model was generated according to conventional marching cube algorithms, followed by automated surface mesh model generation. The two-year mesh model was superimposed on the 6-month mesh model and rigidly aligned by anatomical landmarks with the help of software for the overlapping of digital images (Geomagic Studio, Morrisville, NC, USA). The distance between the 2 surface meshes was presented as color-coded graded figures to identify zones of facial bone resorption (Figure 25). The overlapping of digital images confirmed the hard tissue stability along time, with little or no bone resorption (Figure 26).

## 3. Discussion

The outcomes of implants installed into grafted areas have been described in a series of studies and reviews, suggesting that augmentation techniques may yield similar implant survival compared to the survival in pristine bone [6, 8, 12, 19–22]. However, only a few patients were included in these studies, with relatively short follow-up periods. In addition, the quality of the grafted bone is still not well understood, and the use of grafting materials or socket augmentation might change the proportion of vital bone in comparison to sockets allowed to heal without grafting [6, 8, 12, 19–22]. Whether these changes in bone quality will influence implant success and peri-implant tissue stability remains as yet unknown, as a reduction of quality of the bone may be detrimental for the long-term outcome of implant treatment in grafted sites of the anterior maxilla [21, 22]. In fact, in the last years, the aesthetic outcome has become the main focus of interest in aesthetically sensitive areas [4, 5, 7, 9, 10, 12], posing a challenge for dental clinicians. The aesthetics of a smile is determined by the characteristics of the teeth and by the harmonious architecture of the soft tissue contours [4, 5, 7, 9, 10, 12]. Patient satisfaction is a key factor in the success of implant therapy and a successful implant must provide an acceptable aesthetic appearance [4, 5, 7, 12]. The aesthetic success of implant-supported restorations can be influenced by several critical factors: some of these are patient-dependent (such as the quality and quantity of hard and soft tissues), while others are clinician-dependent (namely, implant positioning, soft tissue management, appropriate prosthetic procedures) [7, 8]. Specifically, the following prerequisites are considered essential for achieving an optimal aesthetic outcome: diagnosis and treatment planning, surgical technique, optimal 3D implant position, ideal implant-abutment design, and emergency profile [7, 8]. According to Raes et al. [12] to achieve an adequate aesthetic result in anterior maxilla with dental implants, favourable periodontal tissue and bone conditions should be present. In fact, implant therapy can be complex, due to numerous local anatomic or traumatic factors resulting in aesthetic commitment in the maxilla. These factors involve thin gingival biotype, thin buccal bone wall, bone dehiscence, and absence of soft and hard tissue quality and quantity, which hamper the success of aesthetic outcomes [12]. An accurate evaluation of all these factors is important for informed decision making and comprehensive treatment planning, with provisions for possible solutions to the expected complications of prosthetic rehabilitation [9, 10, 13]. Recently, CBCT units have been developed for accurate 3D evaluation of the hard tissues in the maxillofacial area, offering advantages such as reduced effective radiation doses, shorter acquisition scan times, easier imaging, and lower costs versus conventional CT methods [9, 10, 13]. In particular, CBCT technology with the smallest FOV is recommended for accurate 3D diagnostics, with a much lower effective radiation dose when compared with multislice CT [9, 10, 13]. In the present study, accurate diagnosis and proper risk assessment resulted in the temporal separation of bone augmentation and implant placement procedures, in accordance with evidence emerging from current literature [9, 10, 12, 13]. In fact, even if the patient was in good systemic health and with a thick gingival biotype, the measurement of buccal and palatal bone plate thickness around the failing tooth using CBCT images revealed the presence of an advanced, localized bone resorption, which renders the simultaneous placement of an implant unpredictable. According to a recent systematic review [22] where the authors showed that delayed implants may be at lower risk of implant failure in reconstructed alveolar ridges, ridge reconstruction with delayed implant placement was selected as treatment option. For this reason, the tooth was gently extracted, and the socket was debrided using alveolar surgical curettes to remove the granulation tissue. After that, a synthetic BCP block was placed into the alveolar socket. In fact, the use of bone substitute preserves the alveolar ridge by stabilizing the blood clot, thus maintaining the volume at the site and simultaneously serving as an osteoconductive guide rail to facilitate new bone formation. In addition, the residual circumferential gap was completely filled with granules of the same material, mixed with tetracycline powder, to obtain a local antibiotic effect. BCP is a synthetic, biocompatible material characterised by high porosity [15–17]. This feature provides adequate space for vascular invasion and angiogenesis. In addition, its microstructure promotes optimal proliferation of osteoblasts, so that particles can easily integrate with the newly formed bone [15–17]. The slow resorption rate of BCP stabilizes the structure of the newly formed bone, to maintain a good volume in the long term [16]. In our present case, BCP was associated with a collagen membrane, in order to restore the ridge shape and dimension and prevent the migration of epithelial and connective cells to the area. After six months of uneventful healing, the implant was placed. A bone core biopsy was retrieved at the site of implant placement, using a trephine bur, to histologically assess the bone quality and structure. The histological evaluation showed extensive new bone formation, embedded with residual particles of grafting material. Bone quality is as critical as bone quantity in determining the long-term function and stability of dental implants and the peri-implant tissues [22]. Accordingly, a major concern with the use of grafting materials is the presence of residual particles. In our present study, the primary stability of the implant was apically searched in the residual native bone, and the major axis was placed palatally, in correspondence with the cingulum, in order to keep a safe distance from the aesthetically sensitive, reconstructed buccal wall. To achieve optimal aesthetic success, it is suggested to place an implant in an ideal 3D position, in order to maintain an adequate amount of buccal bone [9, 10, 12]. In fact, when an implant is placed too facially, a resorption of the buccal bone wall may occur, with a subsequent recession and unpleasant aesthetic outcome [9, 10, 12]. Moreover, care must be taken in the mesiodistal remaining space between the implant and the adjacent teeth: since the formation of the papilla depends on the underlying bone support, a minimum of a 1.5 mm space between the implant and the adjacent teeth must be left, for the correct maturation of the papilla [9, 10, 12]. As emerging in the current literature, single implants in the anterior maxilla may be early restored/loaded, with predictable osseointegration and high implant survival rates [2, 4, 5, 12]; in this context, adequate primary implant stability and avoidance of occlusal or eccentric contact during the healing period are considered prerequisites for success. In the present case, the optimal initial implant stability allowed the early restoration/loading of the implant, with the benefits of optimal gingival contour before definitive prosthesis, shortened treatment time, patient satisfaction, and fewer surgical interventions. In fact, three weeks after placement, the patient was provided with a provisional restoration. The patient was instructed to maintain good oral hygiene by brushing and flossing. After waiting for another 3 months, the patient has a good soft tissue maturation induced by the design of the provisional crown; thus the definitive, ceramo-metallic restoration was delivered, with good accuracy as well as a proper emergence profile to support the tissue. The patient was instructed to maintain good oral hygiene by brushing and flossing. Two years later, the patient was visited again to verify overall the aesthetic, functional, and radiographic integration of the implant. The implant-supported restoration was aesthetically integrated and in function, with pleasing soft tissue contours and no clinical issues: clinical examination showed absence of gingival recession, no probing pocket depths, and no bleeding on probing or suppuration. The radiographic examination by means of small FOV CBCT confirmed the hard tissue stability along time, with little or no bone resorption. Finally, in order to better understand the modifications that occurred at the bone crest level around the implant along time, a novel 3D method utilizing digital model superimpositions based on 2 consecutive CBCTs (CBCT at implant placement versus CBCT 2 years after implant placement) was used [9]. This novel 3D analysis allowed for the characterization of dimensional alterations of the facial bone wall in the aesthetic zone along time. The bone crest around the implant was stable along time, with little or no variations. Interestingly, as previously reported [9], central and proximal areas of the facial bone wall displayed a different bone resorption pattern: a risk zone more susceptible to bone loss was identified in the central area. In the present study, a Morse taper connection implant was used to restore the single-tooth gap in the aesthetic area of the anterior maxilla. This implant is characterized by a cone Morse taper interference-fit (TIF) locking-taper, combined with an internal hexagon; the Morse taper presents a taper angle of 1.5° [4, 5, 23, 24]. This type of implant-abutment connection is characterised by high mechanical stability, and it can avoid micromovements at the implant-abutment interface, as demonstrated by several studies [24–26]. In addition, with Morse taper connection implants, the abutment and the fixture behave as a single piece, due to a “cold-welding” effect: there is no microgap between the implant and the abutment and therefore no bacterial leakage, reducing the level of peri-implant tissue inflammation to a minimum [24–27]. In implants with screw-retained abutments, in fact, the microgap of variable dimensions (40–100 *μ*m) between the implant and the abutment can be colonized by bacteria, potentially generating a chemotactic stimulus sustaining the recruitment of inflammatory cells, ultimately resulting in inflammation and osteolysis [28, 29]. The high mechanical stability of the Morse taper implant-abutment assembly, together with the absence of microgap, may effectively protect the crestal bone around implants removing two potential reasons for crestal bone loss [24–27]. Finally, with a tapered interference fit, the abutment emergence geometry gives “platform switching” advantages [4, 5, 23, 24, 30], with increased space for connective tissue, improving the biological seal. This space can guarantee excellent soft tissue healing, with a thicker, larger, well-organized volume of peri-implant soft tissues, protecting the bone crest from resorption [4, 5, 23, 24].

## 4. Conclusions

Today, aesthetics poses a challenge in clinical practice and is critical for successful implant-supported restoration in the anterior maxilla. Our case consisted of a severely compromised maxillary central incisor tooth due to localized advanced chronic periodontitis. Our goal was to obtain aesthetically pleasing soft tissue contours. Based on clinical and radiographic examinations, tooth extraction followed by reconstructive procedures and delayed implant placement was proposed and accepted by the patient. To reconstruct the deficient ridge, a GBR technique was employed, with a BCP block graft placed in the extraction socket in conjunction with granules of the same material and a resorbable barrier membrane. This ridge reconstruction technique enabled us to preserve both hard and soft tissues and counteract vertical and horizontal bone resorption after tooth extraction and allowed for an ideal 3D implant placement. In conclusion, localized severe alveolar bone resorption of the anterior maxilla associated with chronic periodontal disease can be successfully treated by means of ridge reconstruction with GBR and delayed implant insertion. The placement of an early-loaded, Morse taper connection implant in the grafted site was effective in creating an excellent clinical aesthetic result and to maintain it along time.
